# Rotavirus gastroenteritis in Pakistan, 2018: updated disease burden

**DOI:** 10.1186/s12879-021-06123-6

**Published:** 2021-05-06

**Authors:** Nosheen Basharat, Asma Sadiq, Muhammad Dawood, Shahid Ali, Alam Khan, Rooh Ullah, Hayat Khan, Aamir Aziz, Hamid Ali, Aamer Ali Shah, Ijaz Ali, Jadoon Khan

**Affiliations:** 1 Molecular Virology Laboratory, Department of Biosciences, COMSATS University, Islamabad, Pakistan; 2 Department of Microbiology and Medical Laboratory Technology, COMWAVE Institute, Islamabad, Pakistan; 3 Department of Microbiology, Quaid-i-Azam University, Islamabad, Pakistan; 4 Department of Microbiology, University of Swabi, Khyber Pakhtoonkhwa, Pakistan; 5 Institute of Biological Sciences, Sarhad University, Peshawar, Pakistan

**Keywords:** Rotavirus, Surveillance, Disease burden, ELISA, RT-PCR

## Abstract

**Objective:**

Rotavirus A (RVA) is a significant cause of severe diarrheal illness and one of the common causes of death in children under the age of five. This study was aimed at detecting the prevalence of RVA in Pakistan after rotavirus vaccines were introduced. Fecal samples were obtained from 813 children from different hospitals in Rawalpindi and Islamabad, Pakistan, from January 2018 to December 2018. To obtain additional information from the parents / guardians of the children, a standard questionnaire was used.

**Results:**

Using an enzyme-linked immunosorbent assay kit (ELISA), rotavirus antigen was detected and ELISA positive samples were subjected to reverse transcription PCR (RT-PCR). The findings showed 22% prevalence of RVA in children with acute gastroenteritis (AGE) via ELISA and 21% prevalence via RT-PCR in children with AGE. There was no statistically significant difference between gender, age and RVA infections. The winter, spring and fall/autumn seasons were statistically significant for RVA prevalence.

**Conclusion:**

The present study will provide post vaccine prevalence data for the health policy makers. The implementation of rotavirus vaccines, along with adequate nutrition for babies, clean water supply and maternal hygienic activities during infant feeding, is recommended. Furthermore, continuous surveillance is mandatory in the whole country to calculate the disease burden caused by RVA.

## Introduction

Rotavirus A (RVA) is one of the leading agents among all the AGE-causing pathogens, account for approximately 200,000 deaths each year among children under 5 years of age in underdeveloped nations [1]. It has also been estimated that about half (49%) of these RVA fatalities occurred in four developing countries, one of which is Pakistan with rotavirus infants mortality rate 67.6 per 100,000 children [2]. Four live-attenuated RVA vaccines (Rotarix™, RotaTeq™, Rotavac™ and RotaSiil™) have received prequalification from the World Health Organization (WHO) and are currently available worldwide, and over 100 countries have implemented one of these vaccines in their routine vaccination programs [3]. Rotaviruses are members of the double stranded RNA family Reoviridae and are categorized into 9 confirmed (A-I) and one tentative species (J) on the basis of distinctive antigenic properties [4]. The 70–75 nm diameter of the non-enveloped triple-layered viral particle has 11 segmented double-stranded RNA (dsRNA) genes, coding six structural (VP1-VP4, VP6, VP7) and five or six non-structural proteins (NSP1-NSP5 or NSP6) based on the strain [5].

In 2009, the World Health Organization (WHO) proposed the implementation of rotavirus vaccines in routine vaccination systems worldwide and, in particular, in countries with a high incidence of diarrhoea-related mortality below 5 years of age [6]. The post-vaccination monitoring studies showed a substantial reduction in RVA prevalence in the countries where the Rotarix™ vaccine was widely used [7].

The government of Pakistan introduced RVA vaccine (Rotarix™) in the EPI (Expanded program on Immunization) plan of each of the four provinces in 2018 with the support of GAVI, the Vaccine Alliance. The effectiveness of RVA vaccines has yet to be determined in hospital based studies in Pakistan [8]. According to a survey, the RVA vaccine could prevent 3061 deaths per year in Pakistan with the existing routine immunization program, with the highest coverage in the capital city of Islamabad, preventing an additional 1648 deaths per year [9]. In Pakistan, only a few studies have been published on the prevalence of rotavirus infection, so the current study is intended to evaluate the prevalence of rotavirus infection among diarrheal patients admitted to the different health care hospitals in Rawalpindi district.

## Material and methods

### Ethical approval

The said 1 year study 1st January 2018 to 31 December 2018 was conducted from various hospital of capital territory of Pakistan, Molecular Virology Laboratory COMSATS University and Comwave Institute Islamabad. This study was approved by the SUIT (Sarhad University of Science and Information Technology) and PIMS (Pakistan institute of medical sciences) hospital Islamabad, Pakistan “Ethical review committee”. Prior to sample collection an informed consent was obtained from parents /guardians of the children**.** All experiments were performed in accordance with relevant guidelines and regulations.

### Sample collection

A total of 813 fecal samples were collected from various hospitals in Pakistan’s capital territories. Samples were collected from patients under 10 years of age and presented with signs and symptoms of watery diarrhea for 2 to 3 days in accordance with WHO standard case guideline. Patients over the age of ten that had a diarrheal complication that lasted more than 7 days, as well as those that had bloody diarrhea, were removed from the study [10]. Table 1 shows the list of hospitals. A clean, sterile and leak proof plastic container about 1–2 ml or 1–2 g were used for collection of the samples and the sample were transferred to Molecular Virology Laboratory, COMSATS University, Islamabad for further processing.

**Table 1:** Representation of samples collected from hospitals of Capital territories in 2018

Hospital	Patients studied	Percentage
District head quarter hospital Rawalpindi	198	24.3%
Holy family hospital Rawalpindi	160	19.6%
Benazir Bhutto Hospital Rawalpindi	180	22.2%
Pakistan Institute of Medical Sciences Hospital Islamabad	275	33.8%
Total	813	100%

### RVA detection via *ELISA*

A 10% fecal suspension was prepared in 1 ml of PBS (Phosphate buffer Saline) and tested for rotavirus presence using a commercially available enzyme immunoassay (EIA) kit RIDASCREEN manufactured by R-Biopharm (Germany) as instructed by the manufacturer*.*

### RVA RNA extraction and RT-PCR

A QIAamp Viral RNA mini kit by Qiagen (Hilden, Germany) was used to extract the viral RNA. Samples found to be positive for the ELISA test were confirmed for RVA outer capsid genes (VP7 and VP4) by reverse transcription-PCR (RT-PCR). The primers used were (Beg9 and End9) for VP7 and (VP4–1-17F and Con2Deg) for VP4 [8]. The RT-PCR was carried out under the following conditions: initial reverse transcription step (30 min at 45 °C), polymerase activation (95 °C for 5 min), 40 cycles of amplification (denaturation: 30s at 94 °C; annealing: 45 s at 45 °C for VP4 and 45 s at 50 °C for VP7; product extension for 1 min at 72 °C) and final extension of 10 min at 72 °C.

### Gel electrophoresis

The PCR product was validated using 2% agarose gel electrophoresis, stained with ethidium bromide and visualized under ultraviolet illumination.

### Statistical analysis

Statistical analyses were carried out using SPSS (Statistical Package for Social Sciences) software (v21.0) [11]. A chi-square test was used to examine the possible relationship between gender difference, age difference, residence, and seasonal distribution and RVA positive and negative status. Prevalence of RVA was determined by dividing the number of cases by the total number of children examined during the study period. The statistical level of significance was set at*<*0.05.

## Results

### RVA prevalence

A total of 175/813 (22%) were found positive via ELISA. Of the 175 RVA antigen-positive samples, 170/175 (97%) were positive by RT-PCR and the remaining 3% were negative by RT-PCR (Table 2).

**Table 2:** Overall prevalence of rotavirus infection among diarrheal patients in 2018

Characteristics	Rotavirus infection	
	ELISA	PCR
Positive	175 (21.5%)	170 (97%)
Negative	638 (78.5%)	5 (3%)
Total	813	175

### Demographic features of children with RVA diarrhea

Demographic characteristics of 175 RVA positive fecal samples of paediatric diarrheal patients were examined. A total 566/813 (70%) patients were male while 247/813 (30%) were female. Of the 170 samples positive by RT-PCR, 57 are females (33.5%) and 113 are males (66.5%). However the statistical analysis showed that gender was non-significantly associated with rotavirus infection as shown in the Table 3. All the patients were distributed in four age groups as age group 1 (Baby) (patients of age 1 day to 12 months), age group 2 (Toddler) (age above 1 year to 3 years), age group 3 (Pre-school) (above 3 years to 5 years of age) and age group 4 (Grade School) (age above 5 to 10 years). The non-significant statistical results were obtained in case of rotavirus among different age groups as shown in the Table 3 (*p* > 0.05). A total of 228/813 (28%) were from rural population while 585/813 (72%) were from urban population. Among the rural residential population 19/228 (8%) were infected while 151/585 (26%) were found infective among urban population residential. The statistical analysis shows that residence of the patients is significant with reference to rotavirus infection as shown in the Table 3 (*p*<0.05).

**Table 3:** A summary of the sociodemographic characteristics of children with RVA positive and negative gastroenteritis via RT-PCR

Characteristics	RV + ve	RV –ve	***p***-value	Total
	**(*n*** **= 170)**	**(*n*** **= 643)**		**(813) (100%)**
**Gender**				
Male	113 (20%)	453 (80%)	0.32	566 (69.6%
Female	57 (23.1%)	190 (77%)		247 (30.3%)
**Age group**				
Age Group 1 (1–12 months)	114 (19.4%)	473 (81%)	0.95	587 (72.2%)
Age group 2 (12–36 months)	37 (39.4%)	57 (61%)		94 (11.6%)
Age group 3 (3–5 years)	0 (0%)	37 (100%)		37 (4.6%)
Age group 4 (5–10 years)	19 (20%)	76 (80%)		95 (11.7)
**Residence**				
Urban	151 (25.8%)	434 (74.2%)	0.001	585 (72%)
Rural	19 (8.3%)	209 (92%)		228 (28%)
**Seasonality**				
Winter	18 (33.3%)	36 (66.7%)	.02	54 (6.64%)
Spring	57 (16.6%)	285 (83.3%)	.01	342 (42.1%)
Summer	76 (20%)	304 (80%)	.55	380 (46.7%)
Autumn	19 (51.4%)	18 (48.6%)	.0001	37 (4.6%)

*P*<0.05 was regarded as statistically significant

### Seasonality of RVA infection

A total of 54/813 patients were studied during Winter (December 1st to February 28th), 342/813 during Spring (March 1st to May 31th), 380/813 during Summer (June 1st to August 31th) and 37/813 during Autumn (September 1st to November 30th). The total infected patients during each season and there statistical analysis shows that winter, spring and fall/autumn seasons were statistically significant for RVA prevalence (*p*<0.05). However summer season was not significantly correlated with the presence of rotavirus infection (*p* > 0.05) (Table 3).

## Discussion

Rotavirus infection associated diarrhea among children is one of the leading cause of death in developing countries of the world including Pakistan [12]. Besides availing the support of Global Alliance for Vaccine and Immunization (GAVI) and World Health Organization (WHO) in controlling the communicable disease burden like diarrhea, the health system of Pakistan is still not developed. Keeping the above situation in view the current study was designed to evaluate the prevalence of RVA infections among the diarrheal patients of Capital territory of Pakistan during 2018.

In the current study the prevalence of RVA was found 21% via RT-PCR which is comparable with a community based study from Karachi, Pakistan reported 17% of prevalence of RVA among diarrheal patients [13]. This rate of prevalence is lesser than 27% reported from Capital territory of Pakistan by [8] and previous studies from Pakistan reported (29–34%) prevalence rate during 2008–2014 [14–17]. Some of the previous studies reported a higher prevalence rate of 63 and 57% from Faisalabad and Karachi respectively [18, 19].

In the present study the RT-PCR was failed to detect RVA among 5 fecal samples positive through ELISA. There may be several reason behind this such as ELISA false positive results, shipment and storage of the samples, degradation in RNA or mutation in gene that could not be detected through general primers and inclusion of older children as most are negative for rotavirus.

In the current study, the prevalence among rural residential population is 19/228 (8%) while 151/585 (26%) were found to be infected among urban residential populations. There is no difference in vaccine coverage between rural and urban areas. People in urban areas have better access to medical care than those in rural areas. As a result, population density and increased transmission may be to blame for the disparity in prevalence rates between the two regions. A pre-vaccination study in Maputo (Southern Mozambique) showed similar results for higher prevalence in urban areas and lower prevalence in rural areas [20].

Weather patterns have indirect but significant impact on the epidemiology of human rotavirus infections. Low indoor relative humidity and indoor associated conditions crowds can be important factors in the epidemiology of rotavirus disease. Hospitalizations for rotavirus gastroenteritis tend to be more common after cold, dry and warm, rainy months of the year [4]. The said study observed RVA based diarrheal infection throughout the year with significant correlated during fall, winter and autumn. Similar supporting results of circulating RVA throughout the year with peak season during dry months and winter season have already been observed in other study from Pakistan [8, 15–17, 19]. The similar pattern of RVA seasonality is observed in other countries of the world [21–23].

Non-significant gender-wise distribution of RVA infection was found in the said study which is comparable with other studies from various areas of the same country Pakistan [8, 13, 17]. Toddlers were the most infected population with 39.4% in the current study supported by several studies which shows that the children below 4 months of age are protected from severe RVA associated diarrhea by maternal antibodies in breast feeding which might be reason for higher incidence rate of RVA in children having age above 7 months [24]. It may also be suggested that the toddler group was not vaccinated so that the prevalence of RVA was high in this group. In contrast to our study 71.4% of patients having age below 1 years were reported by several studies from Pakistan by [8, 13, 16–18]. The rate of incidence of RVA was low among the children of 3 to 5 years of age which is comparable with sadiq et al., 2019. A similar supporting trends were observed in Bangladesh, India, Vietnam, kenya [25–29]. Low incidence has been seen in older age groups because higher age groups may develop immunity after each infection [8].

This study had many drawbacks, like several studies that were carried out in the past. Pakistan has implemented rotavirus vaccine in its vaccination program in January 2018. In this post vaccination study, RVA genotyping and other causes of diarrhoea among the respondents were not examined and the burden of rotavirus disease from this area is not representative of the burden throughout the country. In future studies, the inclusion of several surveillance sites representing the entire country and the monitoring of RVA genotypes would include the estimation of the RVA-related disease burden and the impact of the implementation of vaccines on the diversity of RVA genotypes.

## Conclusions

To conclude, rotavirus is an important aetiology of acute gastroenteritis in Pakistan among children under 5 years of age. Our study highlights that rotavirus in Pakistan during 2018 causes a high burden of disease in children under 5 years of age. In order to avoid rotavirus infection and to lessen the burden of disease, effective rotavirus vaccination may be considered in Pakistan. It also underscores the importance of hygiene and sanitation in the country. Studies such as this provide evidence that will allow healthcare professionals to establish a more comprehensive diagnostic program that will take into account the potential for viral diseases. Further research to discover the predominant RV genotypes in Pakistan is also required for the future production of vaccines.

## Data Availability

The datasets used and/or analysed during the current study available from the corresponding author on reasonable request.
